# The Effect of Proprotein Convertase Subtilisin/Kexin Type 9 Inhibitors on Nonfasting Remnant Cholesterol in a Real World Population

**DOI:** 10.1155/2018/9194736

**Published:** 2018-07-19

**Authors:** Anthony P. Morise, Jennifer Tennant, Sari D. Holmes, Danyel H. Tacker

**Affiliations:** ^1^Section of Cardiology, West Virginia University Heart and Vascular Institute, Morgantown, WV, USA; ^2^Department of Pathology, Anatomy, and Laboratory Medicine, West Virginia University School of Medicine, Morgantown, WV, USA

## Abstract

**Background:**

Proprotein convertase subtilisin/kexin type 9 (PCSK9) inhibitors have demonstrated significant effects on low-density lipoprotein (LDL) cholesterol and nonhigh density lipoprotein (HDL) cholesterol. To date, there have been limited reports on the effect of PCSK9 inhibitors on remnant cholesterol.

**Objectives:**

Assess the effect of PCSK9 inhibitors on nonfasting remnant cholesterol in a real world population. Identify whether pretreatment triglyceride levels are associated with PCSK9 inhibition success as indicated by changes in remnant cholesterol levels.

**Methods:**

Patients in our adult lipid clinic (*n* = 109) receiving PCSK9 inhibition for atherosclerotic cardiovascular disease or familial hypercholesterolemia who had available pre- and post-PCSK9 inhibition standard nonfasting lipid data were, retrospectively, selected for data analysis. Remnant cholesterol was the difference between non-HDL and LDL cholesterol. LDL cholesterol was measured directly and calculated from Friedewald and Martin/Hopkins methods. Data were analyzed using repeated measures ANOVA and multivariable linear regression for differential effects on remnant and LDL cholesterol based upon pretreatment nonfasting triglyceride levels.

**Results:**

Remnant cholesterol as well as total, LDL, non-HDL cholesterol, and triglycerides decreased significantly (*P*<0.001) after PCSK9 inhibition. Patients with higher pretreatment triglyceride levels showed greater decrease in remnant cholesterol after PCSK9 inhibition (*P*<0.001) than those with lower pretreatment triglycerides.

**Conclusions:**

In patients receiving PCSK9 inhibitors, remnant cholesterol as determined from nonfasting blood was reduced in proportion to pretreatment triglycerides.

## 1. Introduction

Proprotein convertase subtilisin/kexin type 9 (PCSK9) inhibitors have demonstrated significant effects on most lipoprotein particles and their respective cholesterol content. The recently completed Fourier outcomes trial demonstrated significant reductions in low-density lipoprotein (LDL) cholesterol, nonhigh density lipoprotein (HDL) cholesterol, total cholesterol, Apolipoproteins B (Apo B) and A1, triglycerides, and lipoprotein a in a large population of patients with stable coronary disease using evolocumab [1]. In earlier efficacy trials, both alirocumab and evolocumab had been demonstrated to lower concentrations of intermediate density cholesterol (IDL) [2, 3]. The pharmacologic reduction of most particles that include Apo B has shown prognostic benefit [4]. Very low-density lipoprotein (VLDL) remnants, IDL, and chylomicron remnants contain Apo B (either Apo B100 or 48) and are often collectively referred to as remnant cholesterol or triglyceride-rich lipoproteins (TRL). In fasting specimens, directly measured remnant lipoprotein cholesterol has been demonstrated to be associated with increased risk of coronary heart disease and large artery atherosclerotic stroke [5, 6]. In nonfasting specimens, a simpler method to estimate remnant cholesterol content has also been suggested to be an independent causal risk factor for ischemic heart disease [7]. While all patients prescribed PCSK9 inhibitors will have unacceptably high LDL cholesterol, all patients prescribed PCSK9 inhibitors will not necessarily have elevated remnant cholesterol or triglycerides.

To date, there are limited reports on the effect of PCSK9 inhibitors on remnant cholesterol [2, 3]. The purpose of this study was to evaluate the effect of these medications on nonfasting remnant cholesterol in a real world population of patients with FDA- and payer-approved clinical indications for the medication and to confirm that the effect of PCSK9 inhibition on remnant cholesterol is dependent upon the baseline triglyceride level.

## 2. Materials and Methods

### 2.1. Patient Population

All patients were >18 years of age and referred to our adult lipid clinic at the West Virginia University Heart and Vascular Institute because of an unacceptably high LDL cholesterol level. All patients had either atherosclerotic cardiovascular disease (ASCVD) or heterozygous familial hypercholesterolemia (FH) without clinical ASCVD as qualifying diagnoses for approval of PCSK9 inhibitors. The vast majority of patients had some adjustment made to their lipid-lowering therapy upon arrival to the lipid clinic. However, all patients were on a stable regimen of lipid-lowering therapy at the time of enrollment and use of the PCSK9 inhibitor. No changes in lipid-lowering therapy were made during the initiation and follow-up of the PCSK9 inhibitor. All patients received approval from their respective payers for the administration of the particular PCSK9 inhibitor that was acceptable to the payer. Criteria used were the patient's qualifying diagnosis, documented record of lipid-lowering therapy, and current lipid levels on that therapy. All patients had unacceptably high LDL cholesterol levels on the maximal medical therapy they were able to tolerate, meaning that their LDL cholesterol levels were >70 mg/dl for ASCVD and >100 mg/dl for FH. Patients could be on moderate to high-intensity statin therapy, completely statin intolerant on 0 mg of statin, tolerant of small doses of statin, on other nonstatin therapies, or some combination of these. All patients included in this analysis had standard nonfasting lipid laboratory data available just before administration and after the administration of at least 3 doses of the PCSK9 inhibitor. Dosing interval for PCSK9 inhibitors was biweekly. All patients received either evolocumab or alirocumab. Approval to use patient data was obtained from the institutional review board and a waiver of consent was granted.

### 2.2. Laboratory Testing

All patients had nonfasting lipid testing. Standard lipid measurements were performed on ARCHITECT c-analyzers using ARCHITECT reagents (Abbott Diagnostics, Abbott Park, IL, USA) including measured lipid profile components (total cholesterol, HDL cholesterol, and triglycerides) and direct LDL measurements at baseline (pretreatment) and minimally after the third dose of PCSK9 inhibitor. An attempt was also made to obtain Apo B measurements before and after therapy. Total cholesterol measurements were performed using standard enzymatic assay employing coupled cholesterol esterase/cholesterol oxidase reactions and detection of quinoneimine product at 500 nm. HDL cholesterol measurements were performed employing selective dissolution of HDL with proprietary detergent and subsequent coupled cholesterol esterase/cholesterol oxidase reactions as for total cholesterol measurement. Triglycerides measurements were performed using standard enzymatic assay employing coupled lipase/glycerol kinase/glycerol phosphate oxidase reactions and detection of quinoneimine product at 500 nm. Direct LDL cholesterol measurements were performed employing selective dissolution of LDL with proprietary detergent and subsequent coupled cholesterol esterase/cholesterol oxidase reactions as for total cholesterol measurement. Apo B measurements were performed at a reference laboratory using the Tina-quant Apolipoprotein B immunoturbidimetric assay (Roche Diagnostics, Indianapolis, IN, USA).

### 2.3. Calculations

Remnant cholesterol was determined by calculating the difference between non-HDL cholesterol and LDL cholesterol as previously described [7]. Non-HDL cholesterol was determined by calculating the difference between total cholesterol and HDL cholesterol. In addition to the direct measurement, LDL cholesterol was calculated using 2 methods: Friedewald equation (LDL = total cholesterol – HDL cholesterol – (triglycerides/5)) [8] and the recently described Martin/Hopkins method [8]. Because the Friedewald equation and Martin/Hopkins method both fail when triglycerides are > 400 mg/dL, the direct LDL measurement was substituted.

### 2.4. Statistical Analysis

All analyses were performed with SPSS Statistics Version 24.0 (IBM Corp., Armonk, NY).* P* value < 0.05 was considered statistically significant. Continuous variables were presented as mean ± standard deviation (SD) and categorical variables were presented as frequency (percent). Pretreatment to posttreatment lipid measures were compared using paired-samples *t* tests. Pearson correlations were conducted to compare changes in remnant cholesterol with changes in other lipid measures. To examine the impact of pretreatment triglyceride levels on change in remnant cholesterol measures, groups were created above and below the median value of pretreatment triglycerides in this sample (>223 versus ≤223). Changes in LDL and remnant cholesterol by pretreatment triglyceride groups were examined using repeated measures ANOVA. Multivariable linear regression analyses were used to examine the impact of pretreatment triglyceride groups on change in remnant cholesterol measures after adjustment for age, gender, diabetes, ASCVD, any statin use, and type/dose of PCSK9 inhibitor (evolocumab 140 mg, alirocumab 75 mg, and alirocumab 150 mg). Beta and B coefficient values are presented from these models to show the standardized and unstandardized estimate of effect, respectively, in remnant cholesterol change for each factor in the model, although only factors significantly associated with remnant cholesterol change were interpreted. The B coefficients can be used to form the regression equation for each model and represent the estimate of effect for each variable using the original units of measurement, whereas the beta coefficient is standardized and can demonstrate the relative strength of effect amongst all factors in the model regardless of differing units of measurement.

It is possible that the phenomenon of regression to the mean could play a role in the results of this study given that there was only one pretreatment measurement and one posttreatment measurement. Therefore, for the purpose of sensitivity analyses, an equation was used to estimate the regression to the mean effect [9] and doubly robust ANCOVA analyses of the triglyceride group effect on change in remnant cholesterol levels, adjusted again by baseline remnant cholesterol levels, were conducted.

## 3. Results

### 3.1. Patient Population

Between September 2015 and January 2018, 122 patients were treated with PCSK9 inhibitors. At the time of this analysis, 10 patients had not received 3 doses of drug and had not obtained follow-up lipid profiles. Three additional patients failed to get adequate follow-up lipid profiles after beginning medication. Therefore, 109 patients had lipid profile data before and after PCSK9 inhibition, including 62 patients with evolocumab and 47 patients with alirocumab (Table 1). Most patients were treated because of ASCVD (mean age = 65.4 ± 10.4 years). Most with ASCVD had statin intolerance, hypercholesterolemia alone, and coronary artery disease as the principle manifestation and were not on any lipid-lowering therapy. Of the 10% of patients who had familial hypercholesterolemia without ASCVD (mean age = 52.0 ± 15.6 years), most had statin intolerance, pure hypercholesterolemia (*n* = 1 with combined hyperlipidemia) and were not on any lipid-lowering therapy. Diabetes of any type was present in 34% of patients, all with ASCVD.

### 3.2. Basic Lipid Data

With the exception of HDL cholesterol, all lipid measures decreased significantly after PCSK9 inhibition (Table 2). Examining continuous variable change scores, greater absolute decrease in triglycerides after PCSK9 inhibition was significantly correlated with greater absolute decrease in remnant cholesterol when LDL was derived using any of the 3 methods (Friedewald equation [*r* = 0.87,* P* < 0.001], Martin/Hopkins method [*r* = 0.77,* P* < 0.001], and direct measurement [*r* = 0.71,* P* < 0.001]). The absolute decrease in remnant cholesterol was significantly greater when LDL was derived by Martin/Hopkins method than when derived by Friedewald equation (–14.3 versus –11.7,* P* = 0.002) or measured directly (–14.3 versus –10.99,* P* = 0.022).

### 3.3. Impact of Pretreatment Triglyceride Levels

**Figure 1:**
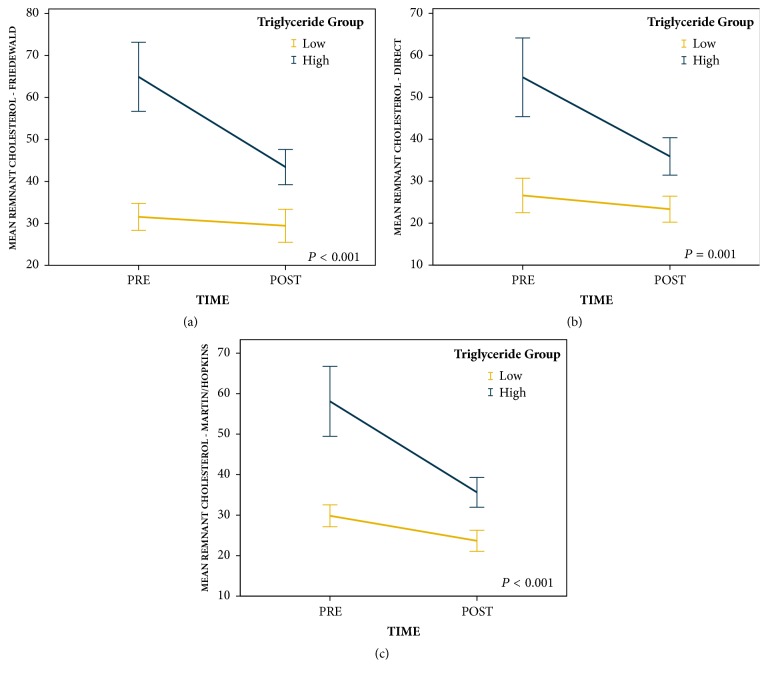
Change in remnant cholesterol levels from pretreatment to posttreatment by pretreatment triglyceride groups (error bars: 95% CI).

**Figure 2:**
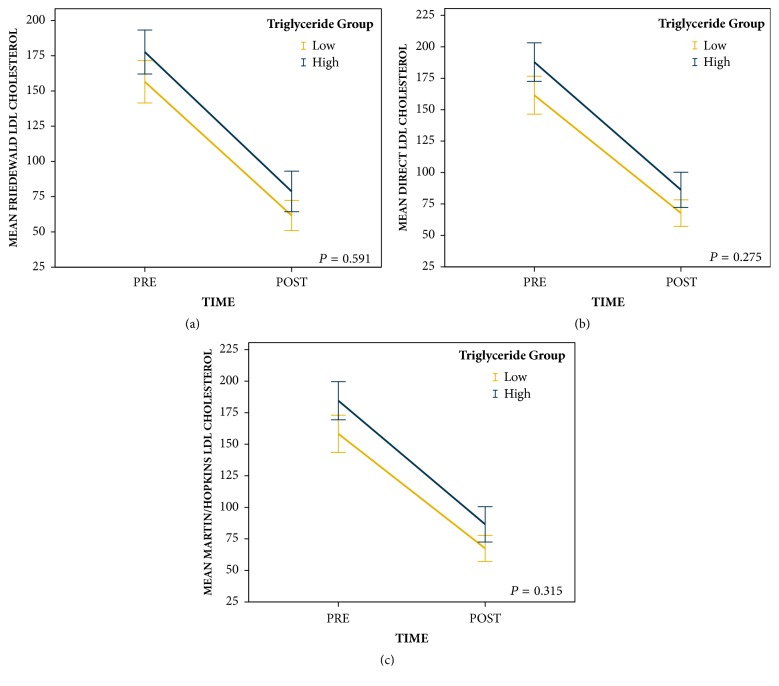
Change in LDL cholesterol levels from pretreatment to posttreatment by pretreatment triglyceride groups (error bars: 95% CI).

Repeated measures ANOVA revealed that patients in the high pretreatment triglyceride group (*n* = 54) had greater reductions in remnant cholesterol than patients in the low pretreatment triglyceride group (*n* = 55) regardless of the way LDL was determined (Friedewald equation [*F* = 20.0,* P* < 0.001], direct measurement [*F* = 11.4,* P* = 0.001], and Martin/Hopkins method [*F* = 16.9,* P* < 0.001]; Figure 1). Repeated measures ANOVA also revealed that patients in the high pretreatment triglyceride group had similar reductions in Friedewald derived LDL (*F* = 0.3,* P* = 0.591), direct measurement LDL (*F* = 1.2,* P* = 0.275), and Martin/Hopkins derived LDL (*F* = 1.0,* P* = 0.315) to patients in the low pretreatment triglyceride group (Figure 2).

In multivariable analyses, pretreatment triglycerides group (low versus high) was a significant independent factor associated with changes in remnant cholesterol after PCSK9 inhibition (Table 3). Specifically, patients in the high pretreatment triglyceride group had greater reductions in remnant cholesterol regardless of the way LDL was determined (Friedewald equation [B = −20.1,* P* < 0.001], Martin/Hopkins method [B = −16.8,* P* < 0.001], and direct measurement [B = −16.3,* P* = 0.001]). Within these multivariable models, the high pretreatment triglyceride group was associated with a 20.1 point, 16.8 point, and 16.3 point greater reduction in remnant cholesterol, respectively, when LDL was determined by Friedewald equation, Martin/Hopkins method, and direct measurement. Multivariable analyses also demonstrated that the type of drug used was significantly associated with changes in remnant cholesterol when LDL was derived with the Friedewald equation and Martin/Hopkins method, but not when direct LDL measurement was utilized. Specifically, patients who received alirocumab 150 mg had greater reductions in remnant cholesterol than patients who received evolocumab 140 mg when LDL was derived by the Friedewald equation (B = −12.1,* P* = 0.036) and Martin/Hopkins method (B = −12.8,* P* = 0.015). Within these multivariable models, alirocumab 150 mg was associated with a 12.1-point and 12.8-point greater reduction in remnant cholesterol compared to evolocumab 140 mg when LDL was derived by Friedewald equation and Martin/Hopkins method, respectively.

The results of sensitivity analysis estimates found that it is possible that the change in remnant cholesterol levels for the low triglyceride group was mostly due to regression to the mean, but the change in remnant cholesterol levels for the high triglyceride group had an effect over and above the effect for regression to the mean. In addition, doubly robust ANCOVA analyses found similar effects for triglyceride group as the primary univariate analyses in the study (Friedewald equation [F = 3.8,* P* = 0.055], direct measurement [F = 4.9,* P* = 0.029], and Martin/Hopkins method [F = 5.3,* P* = 0.023]).

## 4. Discussion

This study demonstrates that remnant cholesterol, like all other Apo B containing lipid fractions, is effectively lowered by PCSK9 inhibition. Importantly, these observations were made in a real world population outside of a clinical trial. In addition, as expected, our findings demonstrate that the degree of remnant cholesterol lowering by PCSK9 inhibition is associated with pretreatment triglyceride concentration, although the same is not true for LDL cholesterol lowering. This finding is consistent with the concept that remnant cholesterol containing larger TRL particles is predicted to be elevated when the serum triglycerides are elevated.

While all of the study patients had approved indications for PCSK9 inhibition, most were not treated with high-intensity statins and thus were considered to be statin intolerant. As a result, pretreatment LDL cholesterol was significantly higher in our study patients than those seen in clinical trials [1]. When compared with the results of the Fourier study [1], the percent decrease in nonHDL, Apo B, and LDL cholesterol in our study was comparable. However, the percent decrease in triglycerides was greater in our study, likely reflecting the nonfasting lipid testing and higher pretreatment triglyceride levels.

We used 3 different methods to generate LDL cholesterol results and then subsequent remnant cholesterol results. Each of the 3 methodologies has strengths and weaknesses. The commonly calculated and nearly universal Friedewald method should not be used when triglycerides are > 400 mg/dL; in such instances, direct LDL measurement is preferred. The recently described Martin/Hopkins method [8] utilizes the same measured variables as the Friedewald method. However, when rapid ultracentrifugation is used as the standard, the Martin/Hopkins method is more accurate than the Friedewald method in estimate of LDL cholesterol especially when the LDL levels are low [10]. Nevertheless, the Martin/Hopkins method still has the limitation when triglycerides are > 400 mg/dL. Direct LDL measurement is not dependent on the measurement of triglycerides for its determination and should not be effected by elevated triglycerides as are the other 2 methods. In fact in the current study, the pretreatment direct LDL was higher than the LDLs estimated by the other 2 methods perhaps reflecting the absence of the triglyceride influence. While directly measured LDL has none of the aforementioned limitations due to triglycerides, it is not as standardized as are the measurements in the standard lipid profile (total cholesterol, HDL, and triglycerides) used to calculate LDL cholesterol. The effect of PCSK9 inhibition on LDL and remnant cholesterol is qualitatively similar using each of the 3 methods. The quantitative differences we may have found in this study will need to be reevaluated using a larger sample size.

Current knowledge concerning how PCSK9 inhibitors work indicates that the interaction between LDL particles and the LDL receptor is effected by PCSK9 such that the LDL receptor (as well as the LDL particle) is ultimately destroyed and never recycled back to the hepatic cellular surface. Remnant particles have a somewhat different pathway for clearance [11]; as the larger TRL particles such as VLDL have their triglyceride content reduced by lipase hydrolysis, they eventually become IDL or smaller VLDL particles. Given sufficient time, they can eventually become LDL particles. Most TRL particles have Apolipoprotein E (Apo E) as well as Apo B on their surfaces, acquired during the process of VLDL production in the liver. Apo E is a ligand for the LDL receptor in the liver as well as for hepatic heparan sulfate proteoglycan receptors (HSPG-R). HSPG-R contain hepatic lipase and polypeptide strands used to capture lipoproteins. Due to configurational issues, many VLDL remnants cannot bind to LDL receptors and must rely on HSPG-R for remnant clearance. Remnant particles become attached to HSPG-R via Apo E binding and undergo further triglyceride lipolysis by hepatic lipase. By this mechanism, many remnant particles diminish in size and become LDL particles suitable for clearance by the LDL receptor. Recent information [12] suggests that, in addition to being involved in remnant metabolism, HSPG-R are PCSK9 receptors that facilitate subsequent PCSK9 and LDL receptor complex formation. Much like the interference with LDL clearance, this PCSK9/LDL receptor complex formation would interfere with the process of remnant clearance as well, due to the reduction in available LDL receptors as VLDL and IDL become LDL particles. Therefore, it would be expected that PCSK9 inhibition by currently available monoclonal antibodies would facilitate the process of remnant as well as LDL clearance.

The differences found by multivariable analysis concerning the effect of equipotent doses of 2 PCSK9 inhibitors on the reduction of remnant cholesterol may be related to sample size or chance alone. Thus, further study in larger groups of patients is needed.

### 4.1. Study Limitations

This study is limited by its lack of a control group, small sample size, lack of specific remnant particle analysis, and direct measurement of remnant cholesterol content. Previously published data involved 81 healthy, normolipemic, nonobese men for evolocumab [2], and 18 healthy volunteers for alirocumab [3]. Evolocumab significantly reduced remnant pool size and increased the fractional catabolic rate of IDL and VLDL [2]. Alirocumab significantly reduced IDL cholesterol content but not VLDL cholesterol content [3]. Larger pools of more clinically relevant patients with appropriate control groups are available from the Fourier [1] (evolocumab,* N* = 27,564), Spire [13] (bococizumab,* N* = 27,438), and Odyssey [14] (alirocumab,* N* = 18,924) outcomes trials. It is unclear whether these large datasets have detailed remnant particle data or directly measured remnant cholesterol as used in prior studies [5, 6, 15]. Nevertheless, the analysis of remnant cholesterol and risk as presented by Varbo et al. [7] suggests that the simple calculation of remnant cholesterol from non-HDL cholesterol minus LDL cholesterol could be sufficient to assess clinical impact. These simpler remnant cholesterol data are available in the 3 large randomized trial populations and could be explored to confirm the findings of our study and determine whether these changes in remnant cholesterol have a prognostic impact independent of the effect on LDL.

We limited our evaluation to nonfasting specimens principally for the sake of patient convenience. However, the use of nonfasting lipids is endorsed by numerous societies, guidelines, and statements including the American Heart Association, the European Society of Cardiology, and the American Association of Clinical Endocrinology [16]. In addition, Varbo et al. [7] have demonstrated through both observational hazard assessments and Mendelian randomization studies that nonfasting remnant cholesterol assessments using the simpler calculation have merit as a means to assess for an effect on clinical outcomes.

### 4.2. Summary

In this real world population receiving PCSK9 inhibition for approved indications and using nonfasting lipid measurements, all Apo B containing particle subgroups had significant reductions in their respective cholesterol concentrations, including remnants. Remnant cholesterol was estimated by the simple subtraction of LDL cholesterol from non-HDL cholesterol. As anticipated, those patients with higher pretreatment triglyceride levels and therefore higher levels of pretreatment remnant cholesterol had a greater decrease in remnant cholesterol after treatment than patients with relatively normal pretreatment triglyceride and remnant levels. Further study in larger populations and with more measurement points will be needed to confirm these findings and determine if these findings will have independent prognostic significance.

## Figures and Tables

**Table 1:** Baseline characteristics of patient sample.

		**Total Sample** ***N* = 109**	
Age (years)		64.1 ± 11.7	
Female		65 (60)	
PCSK9 Inhibitor			
Evolocumab 140mg		62 (57)	
Alirocumab 75mg		23 (21)	
Alirocumab 150 mg		24 (22)	
ASCVD		98 (90)	
Coronary		87	
Peripheral		2	
Cerebral		1	
Polyvascular		2	
Coronary calcium		6	
FH without ASCVD		11 (10)	
Diabetes		37 (34)	
Lipid diagnosis ASCVD			
Hypercholesterolemia alone		61 (56)	
Combined hyperlipidemia		37 (34)	
Therapy	*Statin Intolerance*	*Any Statin*	*Any LLRx*
ASCVD	78 (80)	20 (20)	44 (45)
FH	9 (82)	2 (18)	2 (18)

ASCVD, atherosclerotic cardiovascular disease; FH, familial hypercholesterolemia; LLRx, lipid lowering therapy; PCSK9, proprotein convertase subtilisin kexin 9.

Data presented as frequency (%) or mean ± SD.

**Table 2:** Lipid levels before and after treatment.

	**Pre-treatment**	**Post-treatment**	%** Change**	***P* value**
Total cholesterol	259.6 ± 70.1	151.5 ± 52.7	–41%	<0.001
HDLc	45.1 ± 11.3	46.0 ± 11.8	2%	0.228
NonHDLc	215.1 ± 68.6	106.5 ± 52.4	–50%	<0.001
Triglycerides	255.3 ± 161.7	191.9 ± 99.8	–24%	<0.001
LDLc				
Friedewald equation	167.0 ± 57.2	70.11 ± 47.2	–58%	<0.001
Direct measurement	174.5 ± 57.2	76.9 ± 46.4	–56%	<0.001
Martin/Hopkins method	171.3 ± 56.4	76.9 ± 46.0	–55%	<0.001
Remnant cholesterol				
Friedewald equation LDLc	48.1 ± 28.2	36.4 ± 16.5	–24%	<0.001
Direct measurement LDLc	40.6 ± 29.9	29.6 ± 15.4	–27%	<0.001
Martin/Hopkins method LDLc	43.8 ± 27.2	29.6 ± 13.1	–32%	<0.001
Apolipoprotein B (*n* = 98)	141.4 ± 40.8	74.2 ± 30.5	–48%	<0.001

HDLc = high density lipoprotein cholesterol, LDLc = low density lipoprotein cholesterol.

**Table 3:** Results of the multivariable linear regression analyses for change in remnant cholesterol when LDL was derived by 3 different methodologies.

**Friedewald Equation**	**B**	**Beta**	***P* Value**
Age	–0.21	–0.10	0.708
Female	8.79	0.18	0.074
Diabetes	–5.25	–0.10	0.278
ASCVD	0.87	0.01	0.913
Statin	–2.45	–0.04	0.669
Alirocumab 75mg vs evolocumab 140mg	–8.39	–0.14	0.156
Alirocumab 150mg vs evolocumab 140mg	–12.09	–0.21	0.036
Triglyceride group (high vs low)	–20.06	–0.41	<0.001

**Direct Measurement**			

Age	–0.31	–0.14	0.193
Female	8.39	0.16	0.113
Diabetes	–1.61	–0.03	0.757
ASCVD	2.90	0.04	0.734
Statin	–8.60	–0.14	0.165
Alirocumab 75mg vs evolocumab 140mg	–1.56	–0.03	0.806
Alirocumab 150mg vs evolocumab 140mg	–7.29	–0.12	0.236
Triglyceride group (high vs low)	–16.29	–0.32	0.001

**Martin/Hopkins Method**			

Age	–0.27	–0.14	0.185
Female	8.62	0.19	0.053
Diabetes	–4.16	–0.09	0.342
ASCVD	1.15	0.02	0.872
Statin	–0.87	–0.02	0.866
Alirocumab 75mg vs evolocumab 140mg	–6.04	–0.11	0.259
Alirocumab 150mg vs evolocumab 140mg	–12.76	–0.24	0.015
Triglyceride group (high vs low)	–16.76	–0.38	<0.001

ASCVD = atherosclerotic cardiovascular disease.

## Data Availability

The data used to support the findings of this study are available from the corresponding author upon request.
